# Simulation of liquid hydrocarbon production via n-tetradecane reforming: A renewable energy approach

**DOI:** 10.1371/journal.pone.0341023

**Published:** 2026-02-09

**Authors:** Abolfazl Farajzadeh, Fatemeh Bahadori

**Affiliations:** Chemical Engineering Department, Urmia University of Technology, Urmia, Iran; Guru Nanak College, INDIA

## Abstract

Considering the current worldwide search for renewable energy, n-tetradecane has attracted attention as a candidate for a hydrogen energy carrier and more sighted for light hydrocarbon within the gasoline range of C_5_-C_12_. This study investigates the production of liquid hydrocarbons by n-tetradecane in two stages process: syngas production through reforming of n-tetradecane and Fischer-Tropsch synthesis. The temperature, oxygen-to-carbon (O_2_/C) ratio, reactor length-to-diameter (L/D) ratio, and steam-to-carbon (S/C) ratio are examined in this study as the parameters that influence the reforming of n-tetradecane and superior result of 800ºC, 0.356 O_2_/C, 6 L/D and 2.3 S/C were established to give high conversion and efficient production of syngas. While the yield of the C_5_^+^ hydrocarbon in the Fischer-Tropsch (FT) reactor increased with rising temperature up to 350°C, but the permissible temperature for process is 290°C, based on these, the temperature was set to optimize productivity and longevity of the catalyst. When using n-tetradecane as feed stock, high C_5_^+^ production was resulted because of its suited H_2_/CO ratio of 2.3. The techno-economic assessment highlights the process’s viability at moderate scales, with economic performance strongly influenced by product price and energy integration.

## Introduction

The increasing global consumption of fuel and the fact that world’s stock of fossil fuel is declining at an alarming rate point to a serious energy problem. The dependence on fossil fuels as major resource for industrial development is unsustainable because not only these resources are depleted but also utilization of them produces greenhouse gases that cause global warming and environmental pollution. This influx of fossil fuel-derived carbon disrupts natural carbon cycles, it has severe ecological and health impacts as well as propensity of leading to climate change.

The optimization of energy systems plays a crucial role in addressing the global energy and fuel problem by enhancing efficiency of process [1–3] and equipment performance [4–5] and reducing energy waste [6–7]. In addition; utilizing carbon dioxide as a carbon source in the production of fuel and chemicals, industries can mitigate greenhouse gas emissions and create a sustainable cycle of carbon usage [8–14]. A complimentary system integrated with renewable resources of energy like solar, wind and biomass are one of the best solutions to minimize a country’s reliance on fossil fuels and minimized environmental effects. To this end, stressing these approaches optimizes the balance between conventional and renewable energy systems to achieve a more stable power system.

Bio fuels from sources such as biomass, microorganism algae and waste organic matters also present a much more sustainable method that does not pollute the environment and could also reduce emissions. Switching to green energy is a means to keep ecosystems from getting destroyed, reduce the risks of energy vulnerability, and progress towards greater sustainability.

Renewable energy infrastructures can play a pivotal role in enabling the conversion of oil-rich biomass into valuable liquid hydrocarbons before natural fermentation releases methane and carbon dioxide into the environment. Through coupling solar- and wind-powered systems with decentralized thermochemical upgrading pathways, oil-containing organic materials—such as those rich in long-chain hydrocarbons like n-tetradecane—can be converted into gasoline-range fuels rather than being left to degrade anaerobically. Advances in renewable energy optimization and intelligent control, including optimal PV allocation, hybrid power system design, and machine-learning-based energy management [15–18], demonstrate how high-efficiency renewable systems can supply the stable and controllable power required for such upgrading processes. These developments underline the synergy between renewable energy management, emission prevention, and synthetic fuel production.

The concept for the generation of renewable fuels also complements the recent discoveries made in the reformation of diesel for hydrogen creation as it helps in minimizing CO_2_ emissions and other sustainable systems. The recent developments in diesel reforming for hydrogen production have recently attracted much interest because of their contributions to the creation of sustainable energy platforms. Recent studies, such as that by Lin [19], have combined thermodynamic modeling and experimental investigations to optimize operating conditions—highlighting the role of O₂/Diesel and H₂O/Diesel molar ratios in maximizing hydrogen yield while preventing carbon deposition. In parallel, Li et al. [20] have explored innovative oxidants such as hydrogen peroxide (H₂O₂), demonstrating its dual functionality as an oxygen source and in-situ heat supplier, particularly beneficial under oxygen-limited conditions. Their findings reveal superior hydrogen selectivity and reforming efficiency compared to conventional oxy-steam methods, underscoring the potential of H₂O₂-driven ATR as a novel route for clean hydrogen production. Prior to these developments these efforts, Lee et al. [21] introduced a novel pressurized diesel reforming approach using an advanced PtRu-CGO catalyst, demonstrating high fuel conversion and hydrogen yields under pressurized conditions, with up to 75% efficiency during prolonged operation. Kumar et al. [22] tackled the challenges of catalytic reforming, such as soot formation and catalyst deactivation, by developing a non-catalytic autothermal reformer that showed promising efficiency and carbon conversion rates, with expectations for further improvements. Wang et al. [23] enhanced diesel steam reforming by utilizing Rh/La_2_Ce_2_O_7_ catalysts, which exhibited high oxygen mobility and reduced carbon deposition, achieving 97.5% conversion efficiency. Zazhigalov et al. [24] contributed with a dynamic mathematical model that effectively simulates diesel reforming, including the handling of aromatic compounds, offering insights into reformer optimization.

Liang et al. [25] used machine learning approach to ascertain promising reforming conditions, with temperature and the ratio of steam to carbon as the critical variables that determine hydrogen production. Altogether, these investigations point out the advances in both catalytic and non-catalytic diesel reforming procedures, establishing the applicability of both in hydrogen production and other purposeful applications of clean energy. Tetradecane (C₁₄H₃₀), a long-chain alkane presents in both fossil fuel deposits and renewable sources. If not properly recovered and processed, organic materials containing n-tetradecane and other hydrocarbons can ferment, releasing significant amounts of methane and carbon dioxide. ATR, which combines partial oxidation and steam reforming, efficiently converts hydrocarbons into hydrogen-rich syngas, with minimal coking when optimal oxygen-to-carbon and steam-to-carbon ratios are maintained. Research into the reaction mechanisms has highlighted the role of catalysts, such as platinum (Pt), in boosting hydrogen yield and minimizing by-products like CO₂ [26–28]. These findings are crucial for optimizing reactor designs to enhance hydrogen production in fuel cell and distributed energy systems.

Although hydrogen production from long-chain hydrocarbons such as tetradecane has been widely studied, the direct conversion of n-tetradecane into transportation-range liquid fuels (C₅–C₁₂) via an integrated reforming–synthesis pathway remains largely unaddressed. Most existing works focus exclusively on syngas production or isolated reforming steps without evaluating their impact on downstream liquid-fuel formation. In this study, we present a comprehensive process simulation that couples n-tetradecane reforming with syngas optimization and subsequent hydrocarbon synthesis, providing a level of integration and analysis that has not been reported for this feedstock. Additionally, while the current work does not model renewable power systems, we emphasize that such integrated fuel-production pathways could be further strengthened by future coupling with renewable energy sources (e.g., wind) to upgrade waste plant-derived oils or lipid-based residues into valuable synthetic hydrocarbons rather than allowing these biogenic materials to degrade. This combined focus on integrated process modeling, synthetic fuel formation, and the potential alignment with renewable and circular feedstock strategies defines the novelty of this work.

### Simulation of synthetic fuel Production from Tetradecane

**Fig 1:**
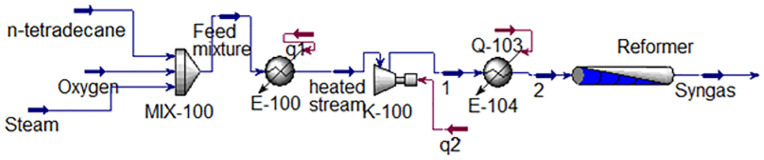
Simulation of n-tetradecane reforming process.

This paper investigates the production of liquid fuel from n-tetradecane. The process has been simulated using Aspen HYSYS. The Peng-Robinson equation of state is implemented to predict the thermodynamic behavior of the materials. Reforming of n-tetradecane has been performed based on the experimental setup of Creaser et al., [28]. Feed enters to the reformer in the operating conditions of 5–11 bar pressure, temperature of 450–850 ºC, 1 < steam/C < 6, and 0.3125 < O₂/C < 0.435. The simulation of n-tetradecane reforming has been shown in Fig 1.

**Fig 2:**
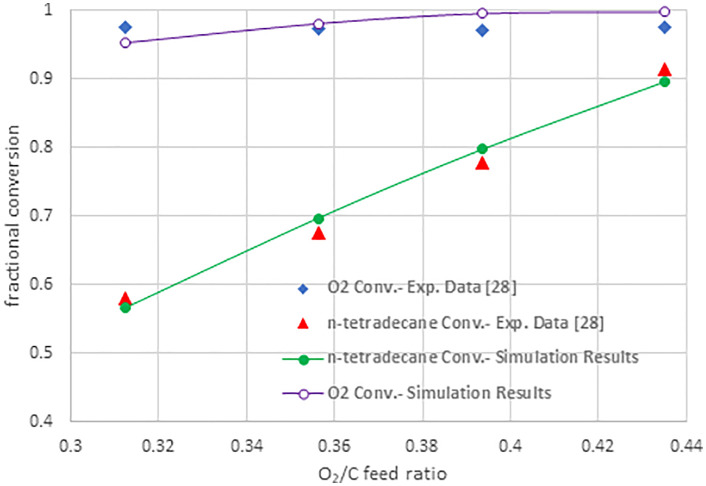
Comparison of simulation results with experimental data.

To validate the simulation results, the conversions of n-tetradecane and oxygen at various O₂/C ratios were compared with the experimental data reported by Creaser et al. [28]. All operating conditions—temperature (450–850 °C), feed O₂/C ratio (0.32–0.43 mol/mol), feed H₂O/C ratio (1.9, 2.4, and 3.0 mol/mol), and gas hourly space velocity (GHSV) of 9700–13000 h ⁻ ¹—were kept consistent with those used by Creaser et al. The reactions were conducted over a Rh-based catalyst. Fig 2 illustrates the comparison between the simulation results and the experimental data reported by Creaser et al. [28]. The average deviations are 2.0% for oxygen conversion and 2.55% for n-tetradecane conversion, demonstrating a strong agreement between the model and experimental data. Based on this agreement, the simulation was further extended to investigate the production of liquid hydrocarbons.

## Proposed method

**Fig 3:**
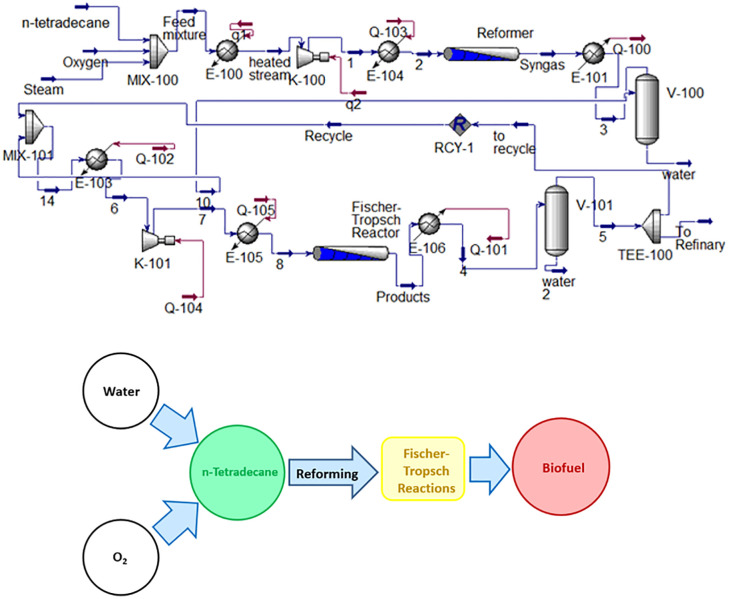
Process for producing liquid hydrocarbons by n-tetradecane reforming.

The process of producing synthetic fuel from n-tetradecane involves two stages: the first stage is the production of syngas from tetradecane, and the second stage is the production of liquid hydrocarbons from the generated syngas. The proposed method for producing syngas from n-tetradecane is autothermal reforming. In this method, n-tetradecane reforms into syngas by steam along with oxygen enter the reforming reactor. After dehydration, the produced syngas is sent to the Fischer-Tropsch reactor. Fig 3 illustrates the proposed process for liquid hydrocarbon production.

The total oxidation reaction, steam reforming, methane formation, and water-gas shift reactions in the presence of a Pt/alumina catalyst have been described as follows [28]:


$$C_{14}H_{30}+21.5O_{2}\to 14CO_{2}+15H_{2}O$$ (1)

$$C_{14}H_{30}+14H_{2}O\to 14CO+29H_{2}$$ (2)

$$C_{14}H_{30}+\frac{13}{3}H_{2}O\to \frac{29}{3}CH_{4}+\frac{13}{3}CO$$ (3)

$$CO+\;H_{2}O\to \;CO_{2}+H_{2}$$ (4)

The reaction kinetics of equations (1)–(4) have been presented as follows [28]:


$$r_{1}=k_{1}y_{tetradecane}^{a}{\times y}_{O2}^{b}$$ (5)

$$r_{2}=k_{2}y_{tetradecane}^{c}{\times y}_{H2O}^{d}$$ (6)

$$r_{3}=k_{3}y_{tetradecane}^{e}{\times y}_{H2O}^{f}$$ (7)

$$\begin{array}{c} r_{4}=k_{4}y_{CO}\times y_{H2O}\left(1-\frac{y_{CO2}y_{H2}}{y_{CO}y_{H2O}K_{e}}\right)where\;K_{e}=\text{exp}\left(\frac{4577.8}{T}-4.33\right) \end{array}$$ (8)

Where, k, K_e_, y, and E_a_ represent pre-exponential coefficient, equilibrium constant, mole fraction, and activation energy, respectively.

The coefficients of the above equations have been reported in Table 1.

**Table 1:** n-tetradecane reforming parameters [28].

Reaction	Parameter	Value	Units
**Fuel total oxidation**	k_m1_	1.44 × 10^−1^	mol kg ^− 1^ s ^− 1^
	E_a1_	108	kJ mol ^− 1^
	a	−0.195	–
	b	0.984	–
**Fuel steam reforming**	k_m2_	3.39	mol kg ^− 1^ s ^− 1^
	E_a2_	116.9	kJ mol ^− 1^
	c	1	–
	d	0.992	–
**Water–gas shift**	k_m4_	6.54 × 10^−2^	mol kg ^− 1^ s ^− 1^
	E_a4_	54.07	kJ mol ^− 1^

The syngas produced during the reforming process is sent to a Fischer-Tropsch reactor for the production of liquid hydrocarbons. The following reactions take place in this reactor [6–8,12]:


$$CO+3H_{2}↔\;{CH}_{4}+\;H_{2}O$$ (9)

$$2CO+4H_{2}↔\;{C_{2}H}_{4}+2H_{2}O$$ (10)

$$2CO+5H_{2}↔\;{C_{2}H}_{6}+2H_{2}O$$ (11)

$$3CO+7H_{2}↔\;{C_{3}H}_{8}+3H_{2}O$$ (12)

$$4CO+9H_{2}↔\;n\_{C_{4}H}_{10}+4H_{2}O$$ (13)

$$4CO+9H_{2}↔\;i\_{C_{4}H}_{10}+4H_{2}O$$ (14)

$$6.05CO+12.23H_{2}↔\;\;{C_{6.05}H}_{12.36}+6.05H_{2}O$$ (15)

$$CO+H_{2}O↔\;{CO}_{2}+H_{2}$$ (16)

Metal-based catalysts such as Fe, Co, Ru, Ni, and Rh have been studied for their performance in Fischer-Tropsch reactions. Due to the high methane selectivity of Ru and Ni-based catalysts, they are not suitable for large-scale industrial applications. Instead, iron and cobalt are commonly employed in industrial settings. Iron catalysts, in particular, are preferred for their favorable activity, lower cost, and ability to operate at temperatures lower than those required for cobalt catalysts. The kinetics of reactions, represented by equations (9)–(16), over iron-based catalysts (100 Fe/5 Cu/ 4.2 K/25 SiO_2_) have been outlined in Bub et al. [29] and Montazer-Rahmati and Bargah-Soleimani [30]:


$$\begin{array}{c} R_{i}=K_{i}\text{exp}\left(\frac{-E_{i}}{RT}\right){P_{CO\;}}^{m}{P_{H2}}^{n} \end{array}$$ (17)

The reactions occurred within a pressure range of 0.3–2.0 MPa.

The related coefficients have been reported in Table 2.

**Table 2:** Kinetic parameters of Fischer-Tropsch reactions [30].

Equation No.	E_i_(j/mol)	K_i_	n	m
7	83400	1.627 × 10^−1^	1.57	−1.08
8	65000	9.571 × 10^−7^	0.07	0.76
9	49800	1.207 × 10^−6^	1.32	−0.56
10	34900	5.841 × 10^−10^	0.66	0.40
11	25700	1.151 × 10^-14^	1.14	0.47
12	25700	5.608 × 10^-12^	0.50	0.82
13	23500	7.806 × 10^-12^	0.60	0.59
14	58800	7.799 × 10^−8^	0.70	0.57

## Results and discussion

The results of the study are divided into two main stages, reflecting the structure of the process itself. The first stage focuses on the reforming process, while the second stage examines the Fischer-Tropsch process. In the entirety of this paper, conversion is defined as follows:

Conversion = (Input n-Tetradecane - Output n-Tetradecane)/ (Input n-Tetradecane) × 100

### Reforming of n-Tetradecane

**Fig 4:**
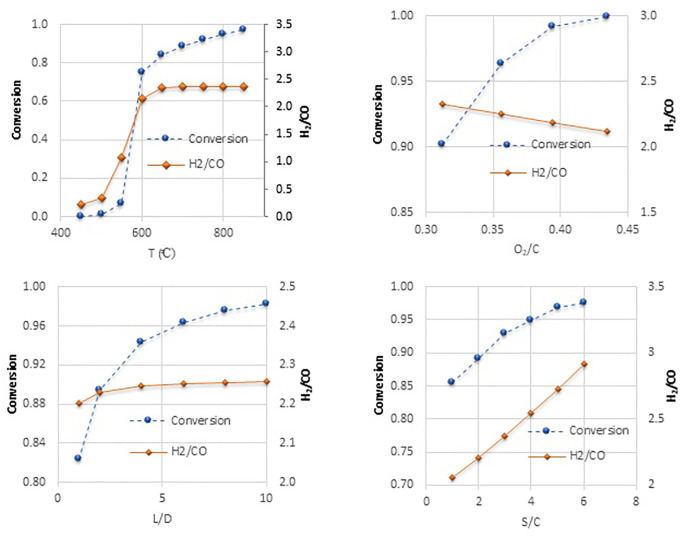
Effects of a) Temperature, b) O_2_/C, c) L/D, and Steam/C on n- tetradecane conversion and outlet H_2_/CO.

The key parameters affecting the reforming of n-tetradecane are temperature, the oxygen-to-carbon reactor length to diameter (L/D), and steam-to-carbon ratios in the feed. The effects of these parameters are illustrated in Fig 4. Fig 4a illustrates the effect of temperature on the conversion of n-tetradecane and the H_2_/CO ratio in the reactor, ranging from 450 to 850 ºC. The conversion remains low at 450–550 ºC, but increases rapidly at 600 due to the heat supplied to the reaction. While the reforming process typically occurs at temperatures up to 1200 ºC, autothermal conditions in the reactor can lead to significant temperature increases. It’s essential to consider the catalyst’s maximum processing temperature; therefore, although higher inlet temperatures can enhance conversion, they are not advisable in an autothermal process. Consequently, the inlet temperature for the reforming reactor was set at 800 ºC based on the conversion and H_2_/CO ratio observed at this temperature.

Fig 4b shows the effect of the O_2_/C ratio on the conversion of n-tetradecane and the exiting H_2_/CO ratio. As indicated, within the O_2_/C range of 0.3125 to 0.435, the H_2_/CO ratio fluctuates between 2 and 2.5, while the conversion rises from 0.9 to 1. This trend can be attributed to two main factors: first, increased oxygen accelerates the oxidation of n-tetradecane, leading to a higher conversion; second, the exothermic nature of the reaction releases heat, providing the activation energy for steam reforming. However, as the oxidation progresses, it also converts n-tetradecane into water and carbon dioxide, which is undesirable for syngas production during steam reforming. Therefore, the O_2_/C ratio must be carefully controlled to maintain acceptable conversion, leading to a setting of 0.356.

Fig 4c shows the effect of the length-to-diameter (L/D) ratio of the reactor on the conversion of n-tetradecane and the exiting H_2_/CO ratio. Most reactions occur at an L/D ratio below 6, with conversion increasing from 0.96 to 0.98 as the ratio rises from 6 to 8. The H_2_/CO ratio remains relatively stable along the reactor’s length. Considering the increased costs associated with longer reactors, the L/D ratio was set at 6 for economic efficiency.

Fig 4d shows the effect of the steam-to-carbon (S/C) ratio on the conversion of n-tetradecane and the exiting H_2_/CO ratio. Most reactions occur at an S/C ratio below 3, with conversion increasing from 0.94 to 0.95 as the ratio rises from 3 to 4. The H_2_/CO ratio varies nearly linearly along the reactor’s length. To balance the higher costs of steam production and reactor volume with economic efficiency, the S/C ratio was set at 2.3.

### Liquid hydrocarbon production in Fischer-Tropsch Reactor

**Fig 5:**
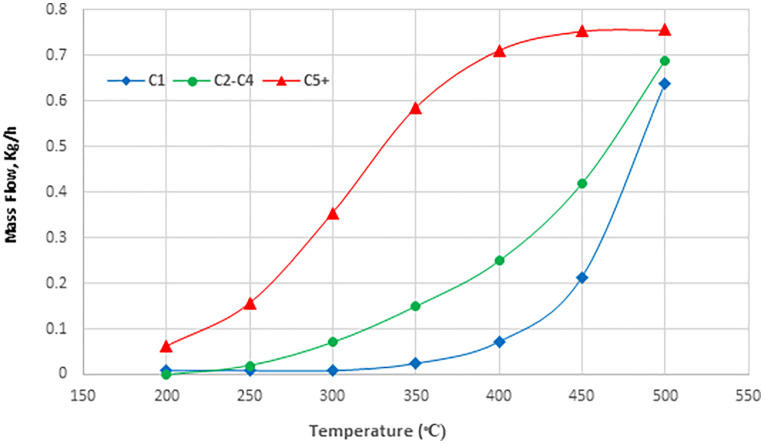
Effect of Temperature on Hydrocarbon Production in the Fischer-Tropsch Reactor.

Fig 5 illustrates the effect of temperature on the Fischer-Tropsch reactor within the range of 200–500°C. The production rates of C_1_ and C_2_-C_4_ hydrocarbons consistently increase with temperature. Conversely, the production rate of C_5_^+^ hydrocarbons rise, peaking at 350°C, before declining. Fischer-Tropsch reactions are exothermic, releasing heat and increasing the reactor’s temperature. Maintaining an optimal temperature is crucial for maximizing hydrocarbon production. At lower temperatures, hydrocarbon production rates increase until they reach a certain point, then begin to decline.

The maximum C_5_^+^ hydrocarbon production is observed within the temperature range of 450°C to 500°C. However, since Fischer–Tropsch reactions are exothermic, the temperature rises along the reactor length. Therefore, maintaining an appropriate operating temperature is crucial to ensure catalyst thermal stability and prolong its lifetime. In addition, as illustrated in Fig 5, the selectivity toward C_5_^+^ hydrocarbons decrease with increasing temperature, underscoring the importance of temperature control to favor the formation of longer-chain hydrocarbons. Based on this analysis, the optimal inlet temperature for maximizing liquid hydrocarbon yield relative to lighter hydrocarbons is determined to be 290°C.

Temperature plays a significant role in reaction conversion, but residence time in Fischer-Tropsch reactors critically influences syngas conversion and hydrocarbon product distribution. Increasing residence time enhances syngas conversion by allowing reactants more time to interact with the catalyst surface. This is particularly important in Fischer-Tropsch reactions, which involve adsorption, surface reactions, and desorption. However, excessively long residence times may trigger secondary reactions, such as the thermal cracking of heavy hydrocarbons, leading to increased light gas production or the formation of undesired products like coke.

Residence time also strongly affects product distribution. At shorter residence times, light hydrocarbons such as methane and other gaseous products (C_1_-C_4_) predominate. As residence time increases, longer hydrocarbon chains form, resulting in higher production of liquid fuels like gasoline and diesel (C_5_-C_11_). Further increases favor heavier products, including waxes and solid hydrocarbons (C_12_^+^), due to extended chain growth. These shifts in product distribution depend on reaction kinetics and operating conditions. Longer residence times may also lower the hydrogen-to-carbon ratio in products, as heavier hydrocarbons have lower hydrogen content.

While longer residence times improve syngas conversion, they may also cause challenges such as carbon deposition on the catalyst, reactor hot spots, and reduced economic efficiency.

### Comparison of liquid hydrocarbon production by different feeds

This section evaluates the effects of different feeds on liquid hydrocarbon production:

1. Methane autothermal reforming [6–7]
2. Ethanol steam reforming (ESR) [12]
3. Ethanol steam reforming + CO2 reforming (ESR + EDR) [12]
4. Ethanol steam reforming + CO_2_ reforming + additional CO_2_ (ESR + EDR + excess CO_2_) [12]
5. n-Tetradecane autothermal reforming

**Fig 6:**
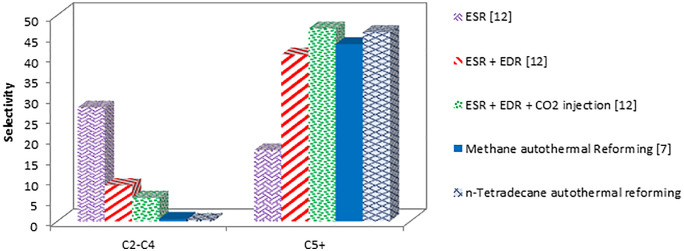
Production rates of C5+ and C2-C4 based on 1 Kg feed.

Fig 6 shows the production rates of C_5_^+^ and C_2_-C_4_ hydrocarbons for these processes. The syngas from n-tetradecane steam reforming produces the highest rate of C_5_^+^ hydrocarbons. This is attributed to the H₂/CO ratio of 2.3 obtained from n-tetradecane autothermal reforming, which is lower than the typical ratio of 5 observed in ethanol steam reforming (ESR) and 3–3.5 for methane steam reforming. Given that the optimal H₂/CO ratio for the production of gasoline-range hydrocarbons via the Fischer–Tropsch process is approximately 2 [6], the 2.3 ratio from n-tetradecane reforming is well-aligned with the process requirements. As a result, it offers enhanced liquid hydrocarbon yields compared to methane steam reforming or methane autothermal reforming, which typically produce higher H₂/CO ratios.

While CO_2_ reforming can improve the H_2_/CO ratio, it poses challenges. Dry reforming cannot occur simultaneously with steam reforming in the same reactor, necessitating separate reactors [8,12]. This separation increases process costs and the likelihood of coke formation.

The production of liquid hydrocarbons from n-tetradecane, using the Fischer-Tropsch (FT) process, shows promise as a viable pathway for renewable fuel generation. In this case, n-tetradecane is used as a feedstock in the FT synthesis, which typically converts syngas into a range of hydrocarbons. While FT processes usually work with syngas derived from coal, natural gas, or biomass, the use of n-tetradecane as a syngas feedstock provides a process by starting with a long-chain alkane. This not only produces syngas generatio but also repurposes a renewable resource that, if left unused, could decompose and release greenhouse gases. While Fischer-Tropsch technology is already established for large-scale synthetic fuel production, utilizing n-tetradecane as a feedstock holds potential for reducing carbon emissions and improving sustainability. However; further research is required to evaluate the scalability, economic feasibility, and environmental benefits of this approach compared to conventional FT processes**.**

### Techno-economic analysis

A comprehensive techno-economic analysis was performed to assess the feasibility of producing synthetic fuels from n-tetradecane derived from biomass waste through autothermal reforming, syngas conditioning, and Fischer–Tropsch synthesis. The entire process was simulated using Aspen HYSYS under steady-state conditions, with mass and energy balances extracted to determine utility requirements, heat duties, and product yields. The process benefits from partial energy self-sufficiency by utilizing light, non-condensable gases generated within the system for internal heating, supplemented by external utilities.

**Revenue** (selling price × production)

**Total Cost** = OPEX + CAPEX

**OPEX** = Oxygen Cost + Steam Cost + Labor Cost

**CAPEX (Annualized)** = Sum of major equipment costs amortized over lifespan (e.g., 10 years at 10% interest)

**Profit** = Annual Revenue – Total Cost

**Break-even point**: where Revenue = Total Cost → profit = 0

Capital expenditures (CAPEX) were estimated using equipment sizing and cost correlations from peer-reviewed literature and publicly available industrial databases. These included major units such as heat exchangers, reformer, compressor, and the Fischer–Tropsch reactor, with all costs adjusted to 2024 USD using standard cost indices. Operating expenditures (OPEX) comprised raw materials—specifically oxygen and steam—labor, water, and electricity. The n-tetradecane feedstock was assumed to be sourced from plant-based agricultural waste, and due to its waste origin, its cost was considered zero in the economic model. Utility prices were based on international averages.

**Fig 7:**
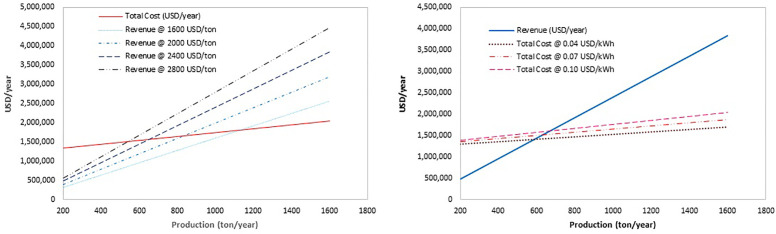
Break-even point analysis based on (a) variation in product price and (b) variation in energy price.

A break-even analysis was conducted to determine the minimum annual production rate required for the process to become economically viable **(**Fig 7). The break-even point was calculated by equating the total annual cost (amortized CAPEX plus OPEX) with revenue from product sales, assuming a base fuel price of 1.2 USD/kg. Sensitivity analyses were also conducted to examine the impact of product price (ranging from 0.8 to 1.6 USD/kg) and energy cost (0.04 to 0.10 USD/kWh) on the break-even point. The results indicate that the selling price of the product has a more significant effect on economic performance than energy costs, emphasizing the importance of product market value and energy integration in the system design. These findings support the process’s potential as a sustainable and economically robust pathway for biofuel production from renewable feedstocks.

It should be noted that the break-even point under baseline assumptions (waste-derived feedstock and internal energy supply) occurs at a much lower production scale. However, when evaluating sensitivity to product price or external energy costs, the break-even point shifts significantly towards higher production levels due to increased total cost or reduced revenue. This highlights the economic vulnerability to market fluctuations and emphasizes the need for cost-stable renewable energy.

### Environmental aspects

Oil-bearing plants and agricultural residues containing long-chain hydrocarbons such as n-tetradecane can undergo microbial degradation if left unmanaged, leading to the release of biogenic methane and carbon dioxide into the atmosphere. Converting these materials into valuable fuels or chemicals therefore offers a circular utilization route that not only recovers energy-rich compounds but also prevents the uncontrolled emission of greenhouse gases. Nonetheless, the associated thermochemical conversion pathways—particularly autothermal reforming followed by Fischer–Tropsch synthesis—demand substantial energy input. This requirement can introduce additional indirect emissions, even when part of the energy is supplied by non-condensable light gases produced within the process itself. To improve the overall environmental performance of such systems, it is advisable to utilize external energy from renewable sources (e.g., wind or solar) and to pursue process-level enhancements such as advanced heat integration, system-wide optimization, and innovative reactor or catalyst designs. These measures can collectively reduce the energy intensity of the pathway and strengthen its viability within a sustainable, low-carbon, circular bioeconomy framework.

## Conclusion

This study successfully demonstrates the potential for producing gasoline-range liquid hydrocarbons (C_5_-C_12_) from n-tetradecane through a two-stage process involving steam reforming and Fischer-Tropsch synthesis. Utilizing Aspen HYSYS for simulation and the Peng-Robinson thermodynamic equation of state for predicting material behavior, we meticulously analyzed operational conditions and their impacts on conversion and product yields.

This study presents an integrated simulation of synthetic fuel production from n-tetradecane, highlighting key process parameters for optimized performance. The main findings are: the optimal O₂/C ratio of 0.356 ensures complete oxygen conversion and efficient n-tetradecane reforming; a reforming temperature of 800°C provides high conversion and an appropriate H₂/CO ratio for downstream synthesis; reactor design parameters of L/D = 6 and S/C = 2.3 balance conversion efficiency with economic feasibility; and a Fischer–Tropsch synthesis temperature of 290°C maximizes the yield of C₅ ⁺ hydrocarbons, enabling efficient production of gasoline-range fuels. These results provide a framework for the rational design and optimization of n-tetradecane-to-liquid fuel processes.

Comparative analysis with other processes, such as ethanol steam reforming and methane autothermal reforming, indicates that n-tetradecane steam reforming achieves a suitable yield of liquid hydrocarbons with a favorable H_2_/CO ratio of 2.3 and remains competitive with other processes.

In addition to the technical analysis, a techno-economic evaluation was conducted under varying product prices and energy cost scenarios, offering insights into the economic robustness and scalability of the proposed process. This enhances the practical relevance of the work in industrial contexts.

Moreover, considering the origin of *n*-tetradecane and similar long-chain hydrocarbons, which can be derived from waste biomass or renewable residues such as used cooking oil, algal lipids, or plant-based oils, the process aligns well with circular bioeconomy principles. Incorporating these renewable feedstocks into the supply chain could significantly reduce the environmental footprint of liquid fuel production and strengthen the case for sustainable synthetic fuel pathways.

In conclusion, this research work offer understanding of how gasoline can be produced from n-tetradecane which serves as a possible solution in the search for renewable energy. Future research should be aimed at fine-tuning of this strategy and its implementation into practice taking into account technological developments and negative environmental effects. Optimizing these processes and utilizing renewable raw material sources would yield major advancements regarding the utilization of fossil fuel and environmental issues.

## Supporting information

S1 Table Break Even point calculations. (PDF)

S2 Table Comparison of production. (PDF)

S3 Table Properties of streams. (PDF)

S4 Table Verification data. (PDF)

S5 Table Results. (PDF)
