# Supplementary material for: Simulation of liquid hydrocarbon production via n-tetradecane reforming: A renewable energy approach
Source: PLoS One. 2026 Feb 9;21(2):e0341023. doi: 10.1371/journal.pone.0341023 (PMC12885370; doi:10.1371/journal.pone.0341023)
Supplement: S1 Table — (PDF) [file pone.0341023.s001.pdf]

S1_Table: Break Even point calculations

kg/h (rounded to 1 decimal)	Annual Production (tons/year)	Total Cost (USD/year)	Revenue @ 1600 USD/ton	Revenue @ 2000 USD/ton	Revenue @ 2400 USD/ton	Revenue @ 2800 USD/ton
22.8	200	1,350,000	320,000	400,000	480,000	560,000
45.7	400	1,450,000	640,000	800,000	960,000	1,120,000
68.5	600	1,550,000	960,000	1,200,000	1,440,000	1,680,000
91.3	800	1,650,000	1,280,000	1,600,000	1,920,000	2,240,000
114.2	1000	1,750,000	1,600,000	2,000,000	2,400,000	2,800,000
137	1200	1,850,000	1,920,000	2,400,000	2,880,000	3,360,000
159.8	1400	1,950,000	2,240,000	2,800,000	3,360,000	3,920,000
182.6	1600	2,050,000	2,560,000	3,200,000	3,840,000	4,480,000

Annual Production (tons/year)	Revenue (USD/year)	Total Cost @ 0.04 USD/kWh	Total Cost @ 0.07 USD/kWh	Total Cost @ 0.10 USD/kWh
200	480,000	1,307,143	1,350,000	1,392,857
400	960,000	1,364,286	1,425,000	1,485,714
600	1,440,000	1,421,429	1,500,000	1,578,571
800	1,920,000	1,478,571	1,575,000	1,671,429
1000	2,400,000	1,535,714	1,650,000	1,764,286
1200	2,880,000	1,592,857	1,725,000	1,857,143
1400	3,360,000	1,650,000	1,800,000	1,950,000
1600	3,840,000	1,707,143	1,875,000	2,042,857
