# Supplementary material for: Simulation of liquid hydrocarbon production via n-tetradecane reforming: A renewable energy approach
Source: PLoS One. 2026 Feb 9;21(2):e0341023. doi: 10.1371/journal.pone.0341023 (PMC12885370; doi:10.1371/journal.pone.0341023)
Supplement: S2 Table — (PDF) [file pone.0341023.s002.pdf]

102 S2_Table: comparison of production

	C2-C4	C5+
ESR (Ahmadi & Bahadori, 2022)	27.56288013	17.2067371
ESR + EDR (Ahmadi & Bahadori, 2022)	8.692708916	40.43154884
ESR + EDR + CO2 injection (Ahmadi & Bahadori, 2022)	5.693679367	46.74163119
Methane autothermal Reforming (Karamlu and Bahadori, 2023)	0.559041513	42.96112649
n-Tetradecane autothermal reforming	0.266520429	45.87386579
