# Supplementary material for: Simulation of liquid hydrocarbon production via n-tetradecane reforming: A renewable energy approach
Source: PLoS One. 2026 Feb 9;21(2):e0341023. doi: 10.1371/journal.pone.0341023 (PMC12885370; doi:10.1371/journal.pone.0341023)
Supplement: S3 Table — (PDF) [file pone.0341023.s003.pdf]

103 S3_Table: Properties of streams

| Stream Name | n-Tetradecane |
|---|---|
| Vapour / Phase Fraction | 0 |
| Temperature [C] | 25 |
| Pressure [bar] | 3 |
| Molar Flow [kgmole/h] | 5.75E-03 |
| Mass Flow [g/min] | 19 |
| Std Ideal Liq Vol Flow [m3/h] | 1.49E-03 |
| Molar Enthalpy [kJ/gmole] | -431.658 |
| Molar Entropy [kJ/kgmole-C] | 612.7441 |
| Heat Flow [kJ/h] | -2480.29 |
| Liq Vol Flow @Std Cond [m3/h] | 1.49E-03 |

| Stream Name | Oxygen |
|---|---|
| Vapour / Phase Fraction | 1 |
| Temperature [C] | 25 |
| Pressure [bar] | 3 |
| Molar Flow [gmole/s] | 0.796 |
| Mass Flow [kg/h] | 91.6992 |
| Std Ideal Liq Vol Flow [m3/h] | 8.06E-02 |
| Molar Enthalpy [kJ/gmole] | -2.82E-02 |
| Molar Entropy [kJ/kgmole-C] | 135.9216 |
| Heat Flow [kJ/h] | -80.8727 |
| Liq Vol Flow @Std Cond [m3/h] | 67.68247 |

| Stream Name | Steam |
|---|---|
| Vapour / Phase Fraction | 0 |
| Temperature [C] | 25 |
| Pressure [bar] | 3 |
| Molar Flow [kgmole/h] | 0.18504 |
| Mass Flow [g/min] | 55.55857 |
| Std Ideal Liq Vol Flow [m3/h] | 3.34E-03 |
| Molar Enthalpy [kJ/gmole] | -286.217 |
| Molar Entropy [kJ/kgmole-C] | 53.70094 |
| Heat Flow [kJ/h] | -52961.7 |

| | |
|---|---|
| Liq Vol Flow @Std Cond [m3/h] | 3.28E-03 |
| Stream Name | heated stream |
| Vapour / Phase Fraction | 1 |
| Temperature [C] | 450 |
| Pressure [bar] | 3 |
| Molar Flow [kgmole/h] | 0.219442 |
| Mass Flow [g/min] | 89.84177 |
| Std Ideal Liq Vol Flow [m3/h] | 5.64E-03 |
| Molar Enthalpy [kJ/gmole] | -193.286 |
| Molar Entropy [kJ/kgmole-C] | 221.5169 |
| Heat Flow [kJ/h] | -42415 |
| Liq Vol Flow @Std Cond [m3/h] | 5.59E-03 |

| | |
|---|---|
| Stream Name | 1 |
| Vapour / Phase Fraction | 1 |
| Temperature [C] | 528.6046 |
| Pressure [bar] | 3 |
| Molar Flow [kgmole/h] | 0.219442 |
| Mass Flow [g/min] | 89.84177 |
| Std Ideal Liq Vol Flow [m3/h] | 5.64E-03 |
| Molar Enthalpy [kJ/gmole] | -189.036 |
| Molar Entropy [kJ/kgmole-C] | 222.858 |
| Heat Flow [kJ/h] | -41482.5 |
| Liq Vol Flow @Std Cond [m3/h] | 5.59E-03 |

| | |
|---|---|
| Stream Name | 2 |
| Vapour / Phase Fraction | 1 |
| Temperature [C] | 800 |
| Pressure [bar] | 5 |
| Molar Flow [kgmole/h] | 0.219442 |
| Mass Flow [g/min] | 89.84177 |
| Std Ideal Liq Vol Flow [m3/h] | 5.64E-03 |
| Molar Enthalpy [kJ/gmole] | -160.798 |
| Molar Entropy [kJ/kgmole-C] | 250.4207 |
| Heat Flow [kJ/h] | -35285.9 |

| | |
|---|---|
| Liq Vol Flow @Std Cond [m3/h] | 5.59E-03 |

| Stream Name | Syngas |
|---|---|
| Vapour / Phase Fraction | 1 |
| Temperature [C] | 1136.259 |
| Pressure [bar] | 5 |
| Molar Flow [kgmole/h] | 0.350329 |
| Mass Flow [g/min] | 89.84949 |
| Std Ideal Liq Vol Flow [m3/h] | 9.52E-03 |
| Molar Enthalpy [kJ/gmole] | -100.709 |
| Molar Entropy [kJ/kgmole-C] | 202.7765 |
| Heat Flow [kJ/h] | -35281.3 |
| Liq Vol Flow @Std Cond [m3/h] | 1.31E-02 |

| | |
|---|---|
| Oxygen | 9.11E-07 |
| CO2 | 6.45E-02 |
| H2O | 0.399545 |
| CO | 0.163394 |
| Hydrogen | 0.371151 |
| C14H30* | 2.63E-05 |
| C5+* | 0 |
| Methane | 1.41E-03 |
| Ethane | 0 |
| Propane | 0 |
| i-Butane | 0 |
| n-Butane | 0 |
| Ethylene | 0 |

| Stream Name | 3 |
|---|---|
| Vapour / Phase Fraction | 0.601787 |
| Temperature [C] | 10 |
| Pressure [bar] | 5 |
| Molar Flow [kgmole/h] | 0.350329 |
| Mass Flow [g/min] | 89.84949 |
| Std Ideal Liq Vol Flow [m3/h] | 9.52E-03 |
| Molar Enthalpy [kJ/gmole] | -158.611 |
| Molar Entropy [kJ/kgmole-C] | 98.74034 |

| | |
|---|---|
| Heat Flow [kJ/h] | -55565.9 |
| Liq Vol Flow @Std Cond [m3/h] | 1.31E-02 |
| Stream Name | 8 |
| Vapour / Phase Fraction | 1 |
| Temperature [C] | 289 |
| Pressure [bar] | 5 |
| Molar Flow [kgmole/h] | 0.210823 |
| Mass Flow [g/min] | 47.91318 |
| Std Ideal Liq Vol Flow [m3/h] | 7.00E-03 |
| Molar Enthalpy [kJ/gmole] | -64.866 |
| Molar Entropy [kJ/kgmole-C] | 151.9905 |
| Heat Flow [kJ/h] | -13675.3 |
| Liq Vol Flow @Std Cond [m3/h] | 4.984197 |

| Stream Name | Products |
|---|---|
| Vapour / Phase Fraction | 1 |
| Temperature [C] | 1521.26 |
| Pressure [bar] | 5 |
| Molar Flow [kgmole/h] | 0.108011 |
| Mass Flow [g/min] | 47.90819 |
| Std Ideal Liq Vol Flow [m3/h] | 3.95E-03 |
| Molar Enthalpy [kJ/gmole] | -126.665 |
| Molar Entropy [kJ/kgmole-C] | 272.3653 |
| Heat Flow [kJ/h] | -13681.2 |
| Liq Vol Flow @Std Cond [m3/h] | 4.03E-03 |

| Stream Name | Products |
|---|---|
| Oxygen | 1.02E-05 |
| CO2 | 0.991807 |
| H2O | 0.984372 |
| CO | 8.76E-02 |
| Hydrogen | 3.67E-02 |
| C14H30* | 3.87E-05 |
| C5+* | 0.766056 |
| Methane | 7.90E-03 |
| Ethane | 3.98E-07 |
| Propane | 1.90E-09 |
| i-Butane | 0 |

| | |
|---|---|
| n-Butane | 0 |
| Ethylene | 4.66E-09 |
