# Supplementary material for: Simulation of liquid hydrocarbon production via n-tetradecane reforming: A renewable energy approach
Source: PLoS One. 2026 Feb 9;21(2):e0341023. doi: 10.1371/journal.pone.0341023 (PMC12885370; doi:10.1371/journal.pone.0341023)
Supplement: S4 Table — (PDF) [file pone.0341023.s004.pdf]

104 S4_Table: Verification data

				n-tetradecane Conv.- Simulation Results	
fuel conversion	O2/C		n-tetradecane Conv.- Exp. Data Creaser et al., 2011	conversion	Error
0.999977415	0.3125		0.58	5.67E-01	2.264452102
0.99997724	0.35625		0.675	6.97E-01	-3.29056536
0.99997709	0.39375		0.778	7.98E-01	-2.53642379
0.999976925	0.435		0.915	8.96E-01	2.121673726
					**2.553**
				O2 Conv.- Simulation Results	
o2 conversion			O2 Conv.- Exp. Data (Creaser et al., 2011)	conversion	Error
			0.975	0.952230043	2.335380187
			0.973	0.980860031	-0.80781411
			0.972	0.99592376	-2.46129218
			0.975	0.99834213	-2.39406462
		0			**2.000**
