# Supplementary material for: Simulation of liquid hydrocarbon production via n-tetradecane reforming: A renewable energy approach
Source: PLoS One. 2026 Feb 9;21(2):e0341023. doi: 10.1371/journal.pone.0341023 (PMC12885370; doi:10.1371/journal.pone.0341023)
Supplement: S5 Table — (PDF) [file pone.0341023.s005.pdf]

105 S5_Table: Results

	Kg/hr				
			Simulation Results		
Temperatue°C	C5+	C1	C2	C3-C4	C2-C4
200	6.22E-02	0.007899739	3.62019E-05	1.98382E-07	3.64E-05
250	1.56E-01	0.007899739	0.000333275	8.45504E-07	0.02012001
300	3.55E-01	0.007899751	0.002811122	3.23845E-06	0.07109228
350	5.86E-01	0.023699253	0.011724251	7.49328E-06	0.14982233
400	7.12E-01	0.071097758	0.029310627	1.0767E-05	0.24897666
450	7.54E-01	0.213293275	0.073276567	1.24271E-05	0.41928373
500	7.57E-01	0.639879825	0.183191419	1.81666E-05	0.68917636
550	7.51E-01	0.853173101	0.512935972	1.85666E-05	0.79917636
600	7.41E-01	0.959819738	1.28233993	1.81666E-05	0.81917636
